# A novel two-dimensional phantom for electrical impedance tomography using 3D printing

**DOI:** 10.1038/s41598-024-52696-y

**Published:** 2024-01-24

**Authors:** Andrew Creegan, Poul M. F. Nielsen, Merryn H. Tawhai

**Affiliations:** 1 Auckland Bioengineering Institute, The University of Auckland, Auckland, 1010 New Zealand; 2 Department of Engineering Science, Faculty of Engineering, The University of Auckland, Auckland, 1010 New Zealand

**Keywords:** Biomedical engineering, Medical imaging

## Abstract

Electrical impedance tomography (EIT) is an imaging method that can be used to image electrical impedance contrasts within various tissues of the body. To support development of EIT measurement systems, a phantom is required that represents the electrical characteristics of the imaging domain. No existing type of EIT phantom combines good performance in all three characteristics of resistivity resolution, spatial resolution, and stability. Here, a novel EIT phantom concept is proposed that uses 3D printed conductive material. Resistivity is controlled using the 3D printing infill percentage parameter, allowing arbitrary resistivity contrasts within the domain to be manufactured automatically. The concept of controlling resistivity through infill percentage is validated, and the manufacturing accuracy is quantified. A method for making electrical connections to the 3D printed material is developed. Finally, a prototype phantom is printed, and a sample EIT analysis is performed. The resulting phantom, printed with an Ultimaker S3, has high reported spatial resolution of 6.9 µm, 6.9 µm, and 2.5 µm for *X, Y*, and *Z* axis directions, respectively (*X* and *Y* being the horizontal axes, and *Z* the vertical). The number of resistivity levels that are manufacturable by varying infill percentage is 15 (calculated by dividing the available range of resistivities by two times the standard deviation of the manufacturing accuracy). This phantom construction technique will allow assessment of the performance of EIT devices under realistic physiological scenarios.

## Introduction

Electrical impedance tomography (EIT) is a medical imaging technology in which tiny electrical currents are applied at the surface of the body, and the resulting surface voltages are measured. Using a tomographic reconstruction algorithm, an image representing the electrical impedance distribution within the body can be created. EIT is particularly useful for imaging the lungs, since the air content in the lungs is strongly correlated with electrical impedance, resulting in relatively high contrast images. EIT can be used to monitor changes in the lungs (e.g., of ventilation and perfusion), or to image pulmonary diseases that affect the air or fluid content, such as chronic obstructive pulmonary disease (COPD) or pulmonary oedema^1^.

Imaging phantoms are used to advance the development of EIT systems. Phantoms provide a well-characterized reference against which EIT measurements can be compared to assess the non-ideal behaviour of a device. This is particularly important in applications that require evaluation of absolute characteristics of lung tissue since this modality of EIT is highly sensitive to measurement errors.

In the literature, there are two types of imaging phantom for EIT that are commonly discussed, each having strengths and weaknesses over a range of characteristics. Salt bath phantoms are those which consist of a bath of conductive fluid (typically a solution of water and NaCl or KCl) with objects placed inside to create conductivity contrasts. Many different styles have been described^2–10^. Various materials have been used as the objects placed in the bath, including gels such as agar, biological materials such as meat and vegetables, and plastics such as acrylic. Salt bath phantoms offer good spatial resolution because the boundary of the bath and the interior objects can be cut to any desired shape. They are also perturbable, i.e., they can be used to measure changes in the imaging domain, because the interior objects can be moved within the bath. However, salt bath phantoms have poor stability because the bathing solution is subject to evaporation, changing its impedance properties.

Discrete element phantoms are those that consist of a fixed layout of discrete electronic components, designed as a physical realization of a finite element mesh^11–13^. Discrete element phantoms provide high accuracy in the impedance of their components, which can be specified precisely, so we consider them to offer high resistivity resolution compared to other types. They are also highly stable. One drawback of discrete element phantoms is their poor spatial resolution. Because they are made up of discrete rather than continuous elements, the spatial information encoded in these phantoms is constrained by the number of elements used. This means they are not as useful as other phantom types for a assessing the spatial characteristics of EIT measurements.

3D printing is a technology that shows promise for a new class of EIT phantoms. Over the last decade, research into 3D printing methods and materials has increased, and several types of electrically conductive 3D printing materials have become available^14–16^. These allow complicated electrically conductive structures to be fabricated easily and with flexibility, using computer-controlled machine execution. Several examples already exist of EIT phantoms made using 3D printed components. Zhang et al.^17^ proposed a skull phantom for EIT partly made from 3D printed conductive material, and de Gelidi et al.^18^ proposed a phantom of the neonatal torso made up of several parts that could be removed to produce a change in internal resistivity. 3D printed parts have good spatial resolution with respect to their manufacturing. The amount of spatial information that they can encode is constrained only by the manufacturing accuracy of the 3D printing process, typically in the range of tens of µm. The benefit of high spatial resolution in a phantom is that it allows for assessment of the performance of an EIT system to an equivalent spatial level. 3D printed phantoms also have good stability in a laboratory environment, being made of plastics.

**Figure 1:**
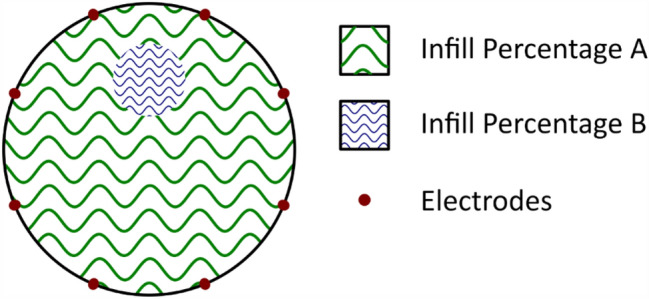
Diagram of 3D printed phantom concept.

In this paper, we propose a fully 3D printed phantom where arbitrarily shaped interior regions of altered resistivity can be manufactured automatically. The key to this process is to vary the resistivity of the 3D printed material by controlling the process parameter known as infill percentage. Figure 1 illustrates this concept.

Much study has been conducted into the effects of the fabrication process on the resistivity of 3D printed parts^19–22^, including the effect of some aspects of infill geometry, such as infill pattern and orientation. However, we are not aware of any study relating resistivity of printed components to the infill percentage, which indicates the ratio of material to air in the 3D printed part. This is relevant to the application of the 3D printed phantom to lung imaging, because the infill percentage is effectively analogous to the air content in lung parenchyma, which has been shown to positively correlate with resistivity^23^.

Table 1 shows a summary of the characteristics of the two existing types of EIT phantom along with the expected characteristics of the proposed 3D printed phantom.

**Table 1:** Comparison of types of EIT phantom.

Characteristic	Salt bath phantom	3D printed phantom	Discrete element phantom
Resistivity resolution	Medium	Medium	Good
Spatial resolution	Good	Good	Bad
Stability	Bad	Good	Good
Perturbability	Good	Bad	Bad
Manufacturability (2D)	Medium	Good	Medium
Manufacturability (3D)	Medium	Good	Bad

In the remaining sections of this paper, the concept of controlling material resistivity by infill percentage is validated, and a numerical relationship between the two is established for a specific material. Manufacturing uncertainty is quantified given a specific set of parameters. To support the practical use of the phantom, two methods are developed for making electrical connections to the 3D printed material: one temporary, and one permanent. Finally, a phantom prototype is constructed and an example EIT analysis is conducted to demonstrate the potential usage of such a phantom.

## Material and printer selection

Table 2 shows a comparison of the resistivities of biological materials, common phantom materials, and Protopasta conductive PLA, which is the conductive 3D printing material that was selected for use here. Blood and air-filled lung are approximately the lowest and highest resistivity materials of the thorax, respectively, that may need to be emulated by an EIT phantom. Physiologic saline and agar—used in salt bath phantoms—have similar resistivity to blood and lung. However, for the proposed 3D printed phantom concept, the bulk material should be lower in resistivity than the materials that will be emulated since decreasing the infill percentage (increasing air “content”) can only result in a higher resistivity than that of the bulk material. Protopasta conductive PLA was therefore chosen to enable matching the resistivity properties of the physiological materials we wish to emulate.

**Table 2:** Comparison of physiological materials and phantom materials.

Material	Resistivity (Ω m)
Lung^24^	7.3–24
Blood^24^	1.5
Physiologic saline^25^	0.83
Agar EIT Phantom^5^	6.67
Protopasta conductive PLA^14^	Bulk material: 0.15 (Printed, *X* and *Y*: 0.30, *Z*: 1.15)

Prints were produced with an Ultimaker S3 3D printer using a 0.4 mm AA print core. The Ultimaker is a fused filament fabrication (FFF) style 3D printer. Ultimaker Cura 5.0.0 was used to prepare sample geometry for printing. Table 3 shows the settings that were used, according to the manufacturer’s specifications for Protopasta Conductive PLA:

**Table 3:** Print settings for Protopasta conductive PLA using Ultimaker Cura 5.0.0.

Setting	Recommended value	Value used
Nozzle temperature [°C]	215	215
Heated bed temperature [°C]	60	60
Print speed [mm/s]	25 to 45	25
Flow rate/extrusion multiplier [%]	100	100
Extrusion width [mm]	0.45 (0.05 mm larger than nozzle size)	0.45
Layer height	–	0.15
Volume flow rate [mm^3^/s]	2–3	1.6

By trial and error, a print speed of 25 mm/s (the minimum of the acceptable range) was selected in order to achieve samples of 100% infill with no voids. A layer height of 0.15 mm was used, though 0.18 mm may have been more appropriate to achieve a volume flow rate within specifications.

**Figure 2:**
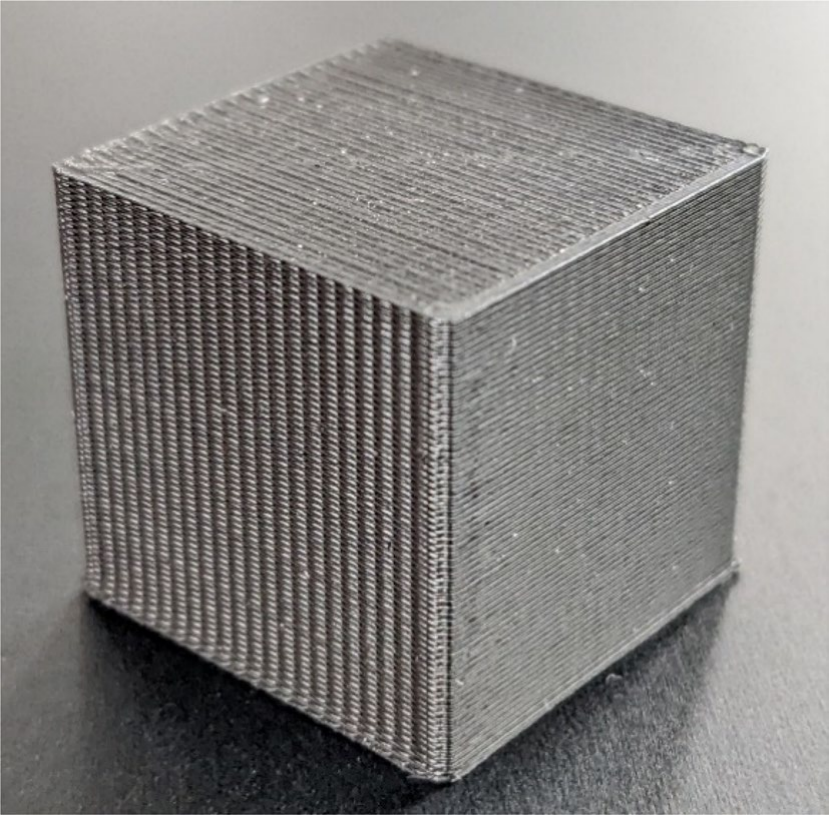
Example of a material test sample of Protopasta conductive PLA. (20 × 20 × 20 mm).

Figure 2 shows a sample of a cube printed with the above settings. The cube’s dimensions are 20 mm × 20 mm × 20 mm.

## Connection methods

The EIT reconstruction problem is poorly conditioned, being more sensitive to resistance contrasts at the domain boundary than those in the centre. Because of this, it is important that the electrode connections be stable, consistent between electrodes, and of low (or at least well-known) resistance. These requirements are also important to accurately characterize the properties of the 3D printed material.

The Protopasta conductive 3D printing filament is a carbon doped polylactic acid (PLA) material. It is highly resistive compared to traditional conductors such as copper (0.15 Ωm versus 1.68*10^–8^ Ωm). Parts printed with FFF have high surface roughness, which is dependent on print orientation and settings. These two factors mean that care must be taken to achieve a high surface area at the electrical connections to this material in order to achieve a low contact resistance.

Accounting for these factors, two connection methods were designed: one for conducting material tests on 3D printed sample cubes, and another for making a permanent connection to the phantom prototype.

### Spring vise

For material sample testing, a vise was constructed to apply constant mechanical contact between the sample and a copper tape electrode backed by rubber. Zhang et al.^20^ used a similar technique, with a silver-copper rubber-based electrode. This is a non-destructive connection method (i.e., it does not alter the sample or leave residue). Thus, by making repeated connections to a single sample, the uncertainty of the measurement process can be assessed as distinct from the uncertainty of the manufacturing process. The drawback of this mechanical contact method is that contact resistance is affected by connection force. Furthermore, excessive contact force has the potential to deform printed samples, especially those printed with low infill percentage ratios.

**Figure 3:**
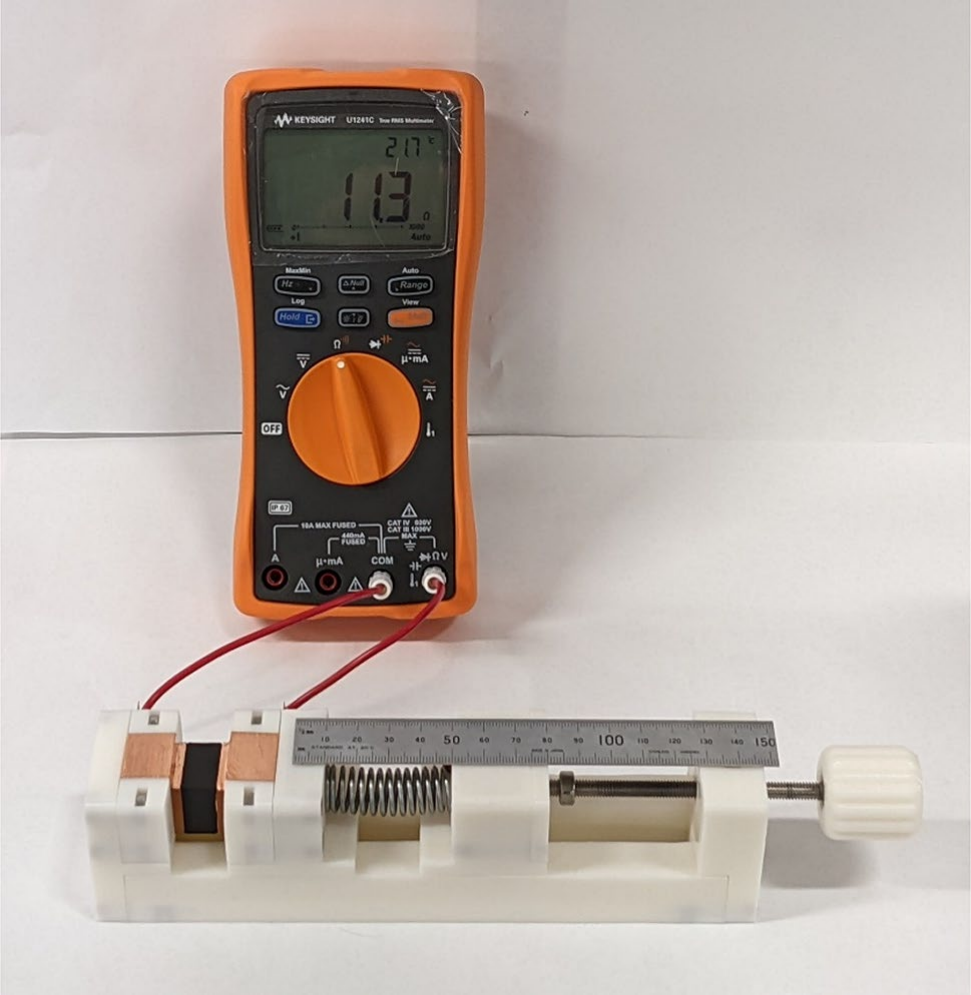
Spring vise designed for repeatable connection to material test samples.

To mitigate these two drawbacks, a spring was placed in line with the jaws of the vise. A precise and repeatable force was achieved by compressing the spring to a set distance, as measured with a ruler. Testing of samples principally consisted of taking a measurement of the sample’s resistance (using a Keysight U1241C digital multimeter). Complex impedance was not measured during this study, though a preliminary measurement with an impedance analyser showed no significant reactive component to the impedance at 20 kHz. Figure 3 shows a sample loaded into the spring vise connected to the Keysight multimeter.

### Spring vise repeatability

The repeatability of the spring vise measurement process was measured. The test was conducted by repeatedly measuring a single material sample. The test specification is shown in Table 4:

**Table 4:** Spring vise repeatability test protocol and results.

Sample		20 mm × 20 mm × 10 mm, 100% Infill (crossed lines pattern)
Test protocol	Measurements	N = 6
	Force (N)	40 (at 40 mm spring length)
	Settling time (s)	60
	Reset time (s)	60
Results	Average resistance (Ω)	5.6
	Standard deviation (Ω)	0.12

For each measurement, the sample was placed in the vise between the two electrodes, and the screw was tightened until the desired spring length was achieved (40 mm, equating to 40 N force). After 1:00 min settling time, the measurement was recorded, the vise was loosened, the sample was removed, and 1:00 min reset time was allowed to elapse before the next measurement. The settling time was designed to be long enough such that a less than 0.1 Ω change was observed on the multimeter in 10 s.

The repeatability of the spring vise measurement process was calculated to be:

1 $${\sigma }_{v}=0.12 \Omega .$$

### Spring vise contact resistance

A model was required for the contact resistance between the spring vise and material samples, along with how the resistance measurement varied with applied force. Three samples of varying length were tested, each at six different force levels. The test protocol is shown in Table 5. The assumptions underlying establishing the contact resistance of the measurement method are the following: (1) the resistance measurement can be modelled by a sample resistance in series with a contact resistance; (2) when multiple samples that are identical except in their length are measured, the resistance measurements should vary linearly with sample length; (3) the contact impedance is assumed to remain constant at a particular applied force and will thus appear as an offset to the linear relationship between resistance and sample length; and (4) the predicted resistance at zero length, i.e., the intercept of the fitted linear model, will be equal to the contact resistance.

**Table 5:** Spring vise contact resistance test protocol.

Samples		3 Samples: 20 mm × 20 mm × {10, 20, 30} mm 100% infill (crossed lines pattern)
Test protocol	Force levels (N)	16, 25, 35, 45, 54, 64
	Settling time (s)	120

**Figure 4:**
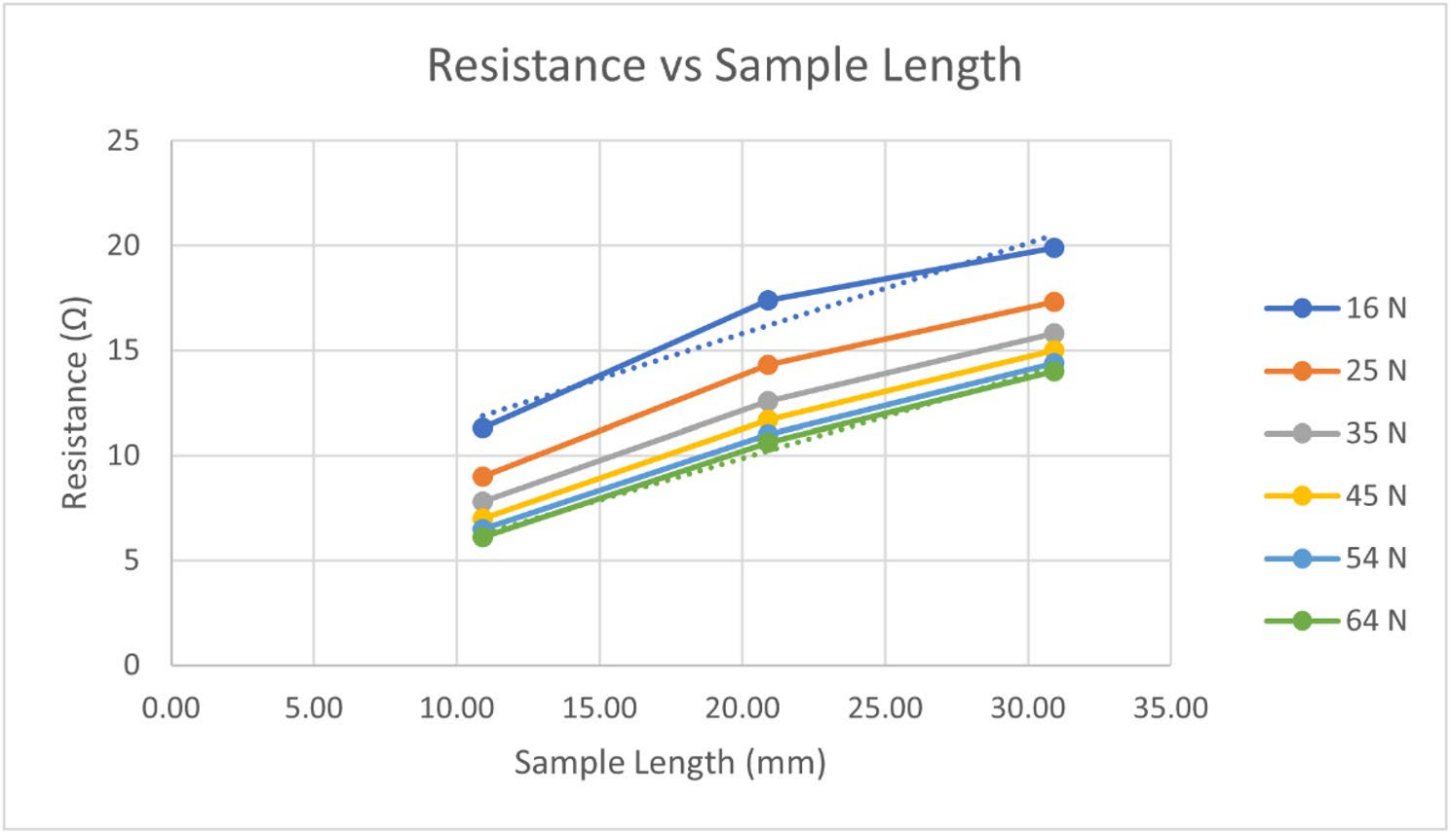
Measured resistance versus length of 3D printed samples for six levels of holding force.

Figure 4 shows the measured resistance versus sample length for all force levels. The assumption that the system can be accurately modelled by a set of samples changing only in length in series with a constant contact resistance would imply a perfectly linear relationship between resistance and length for each force level. If applied force affected only contact resistance, the slope of resistance versus length at each force level would be expected to be the same.

In Fig. 4 we see that the resistance measurement at 20 mm was consistently higher than the fitted linear relationship between resistance and sample length. The deviation from the linear fit decreased with resistance, indicating that the relationship was more linear with higher forces (R^2^ 0.99 at 64 N, R^2^ 0.94 at 16 N). The slopes of the linear relationships were similar for each level of force, decreasing slightly as force increased, which may imply an element of deformation of the samples.

Note that the actual values of the slopes in Fig. 4 are not relevant to determining spring vise contact resistance. See the Infill Percentage vs. Resistivity section for a model of material resistivity.

**Figure 5:**
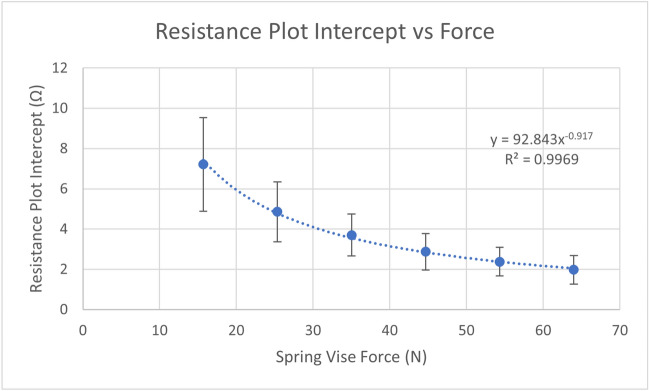
Holding force versus contact resistance for 3D printed samples. Error bars show standard deviation.

Figure 5 shows the intercepts of the linear fits from Fig. 4 plotted versus force. These are the calculated contact resistances at each force level. The error bars represent the standard deviation of the intercept of each linear fit. It is expected that there will be a relationship between contact resistance and applied force, because with increasing force, more of the surface structure of the two materials comes into contact. Figure 5 shows that as applied force increased, contact resistance decreased (in an exponential manner). This suggests that it would be impractical to attempt to obtain a negligible contact resistance. However, we have already shown that the spring vise measurement method is repeatable, therefore characterization of the contact resistance will be sufficient for further study of the printed sample material properties.

The remaining tests use an applied force of 40 N. By linear interpolation, the contact resistance at 40 N was

2 $${R}_{v(40N)}=3.29\Omega .$$

### Silver glue

**Figure 6:**
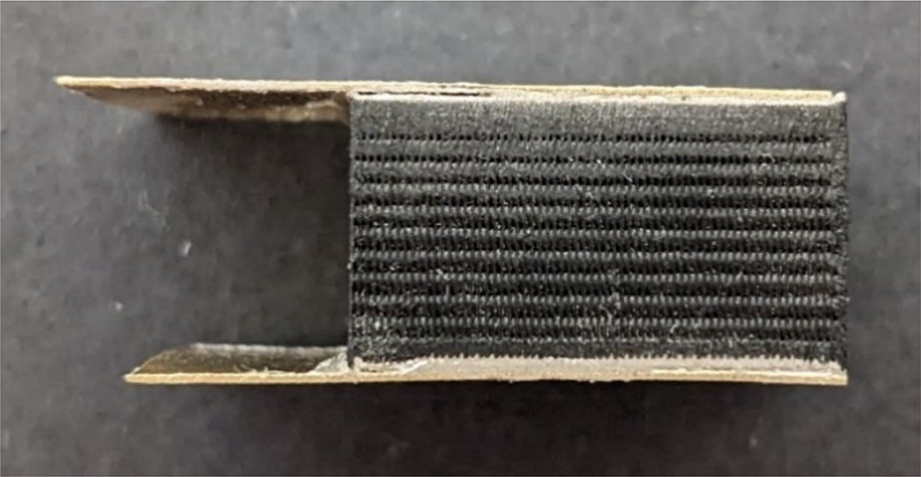
3D printed sample with brass electrodes attached by silver epoxy.

A permanent connection method was required to connect electrodes to the body of a 3D printed phantom. MG Chemicals 8331D Silver Conductive Epoxy Adhesive was selected. A resistance test was conducted, measuring the resistance of nine 3D printed samples with brass electrodes attached by silver epoxy (shown in Fig. 6). Testing of the nine samples resulted in an average measured resistance of 5.6 Ω with a standard deviation of 0.4 Ω.

If we assume that the measured standard deviation of the silver glue samples (*σ*_*measured*_) is a combination of variability in silver glue resistance (*σ*_*silver*_) and variability in sample resistance (*σ*_*sample*_), then the we can obtain the standard deviation of the silver glue resistance by subtracting uncorrelated variances.

$${\sigma }_{silver}=\sqrt{{{\sigma }_{measured}}^{2}-{{\sigma }_{sample}}^{2}.}$$

Similar samples measured with the spring vise were found to have a standard deviation of 0.23 Ω, therefore the repeatability of the silver glue connection method was calculated to be

3 $${\sigma }_{silver}=0.33\Omega .$$

## Material properties testing

To characterize the properties of the 3D printed material, a set of tests was conducted on 3D printed samples.

### Infill percentage versus resistivity

**Figure 7:**
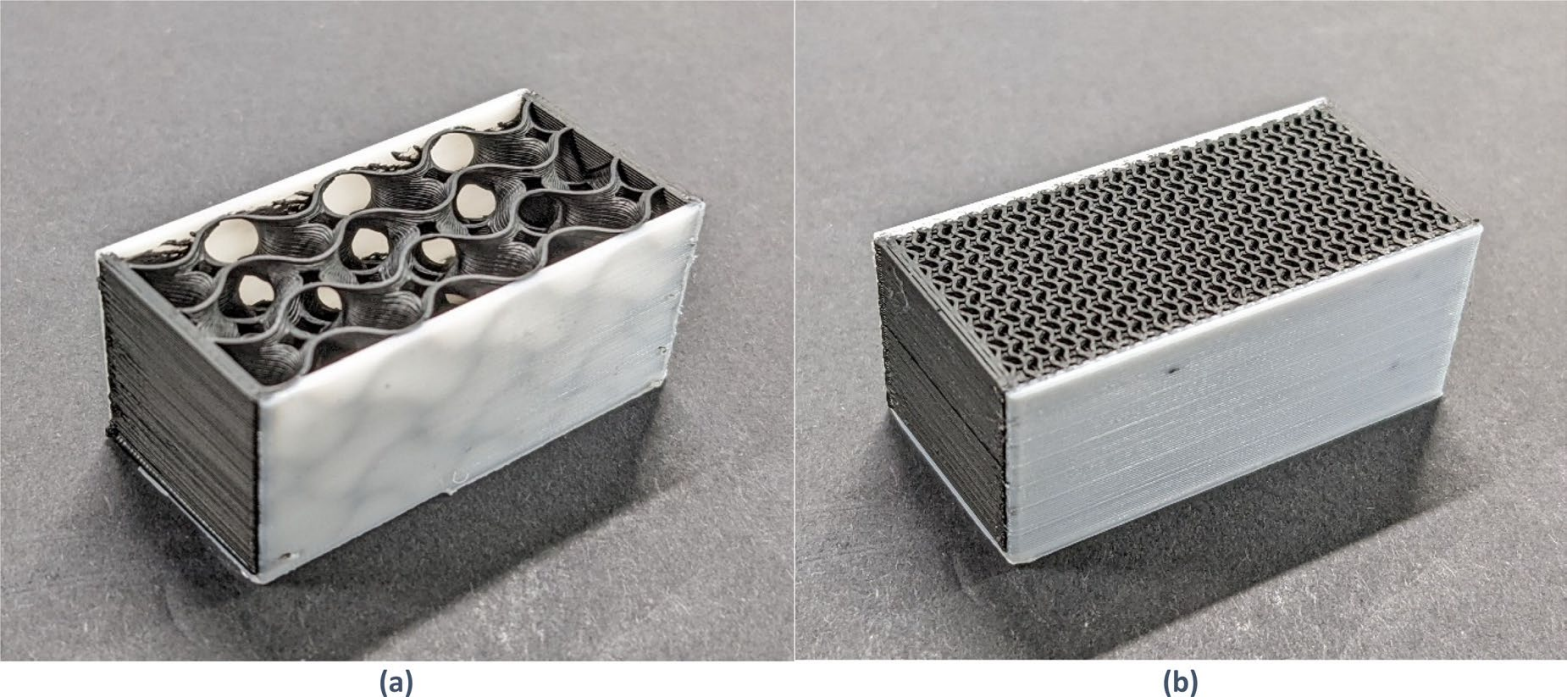
3D printed samples with different infill percentage settings. (**a**) 10%. (**b**) 50%.

The key hypothesis underpinning the proposed phantom concept is that the effective resistivity of a 3D printed conductive material can be controlled by varying the infill percentage setting of the print. To test this, nine different material samples were printed with the infill percentage setting varying from 10 to 90%. Figure 7 shows two of the samples used. The samples were designed so that the resistance measurement would accurately represent the resistance of the infill pattern. This was achieved in the following way: The two ends were printed as a solid cap to maximize contact with the electrodes. The sides were printed in non-conductive white PLA to avoid current flowing through non-infill geometry. A gyroid infill pattern was selected. The gyroid is a triply-periodic geometric pattern that has been proposed to reduce mechanical anisotropy in 3D printed parts^26^. Its suitability for minimizing resistivity anisotropy will require further testing. The test protocol is shown in Table 6.

**Table 6:** Infill percentage versus resistivity test protocol.

Samples		9 Samples: 20 mm × 20 mm × 40 mm. {10, 20, 30, 40, 50, 60, 70, 80, 90} % infill setting (gyroid pattern)
Test protocol	Force (N)	40
	Settling time (s)	60

**Figure 8:**
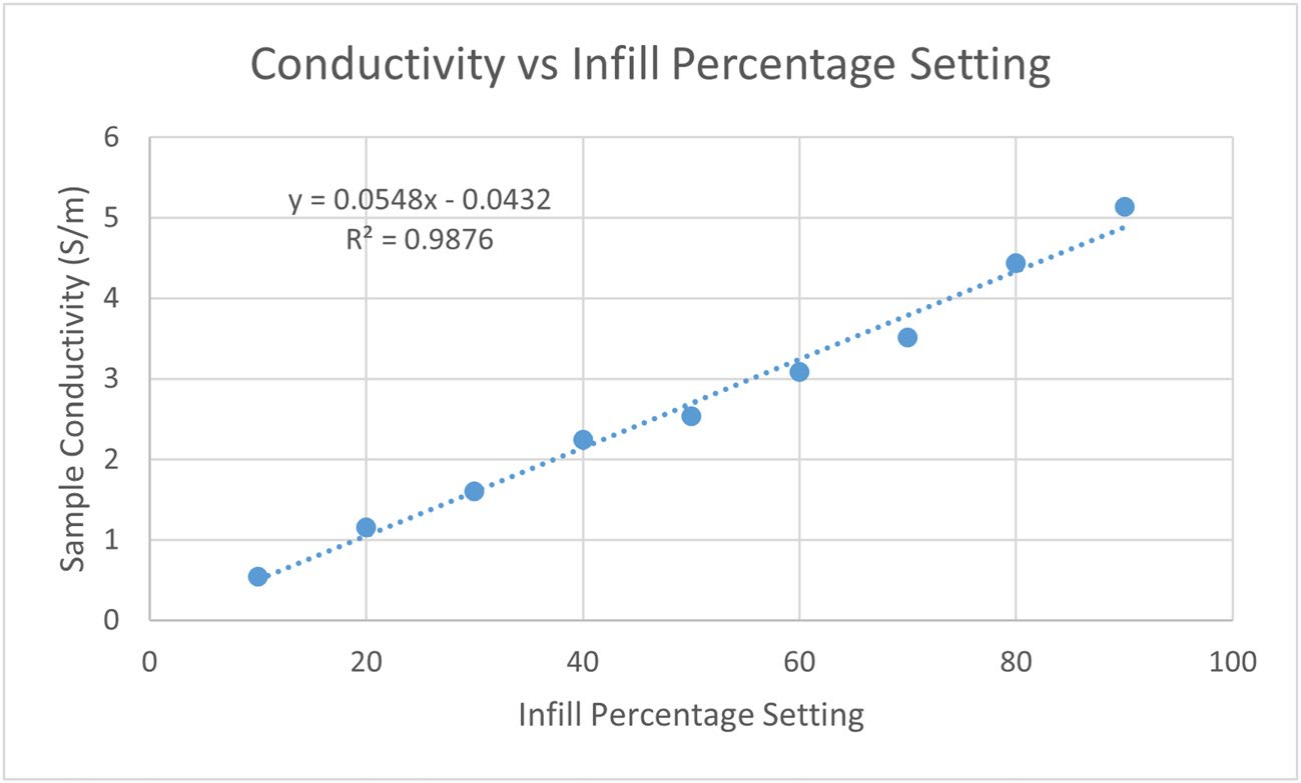
Conductivity versus infill percentage setting of 9 3D printed samples.

Figure 8 shows measured conductivity versus infill percentage setting for the nine infill samples. Conductivity was calculated using measured conductance (the reciprocal of resistance) and the nominal length, width, and height of the samples. Contact resistance and resistance of the end caps were subtracted.

There was a strong linear relationship between measured conductivity and infill percentage setting. This confirms the hypothesis that resistivity can be controlled using this setting. According to the fitted linear relationship, the conductivity at a 100% infill setting would be 5.5 S/m, corresponding to 0.18 Ωm. This is lower than the value of 0.3 Ωm cited in the material’s datasheet for *XY* resistivity of 3D printed objects, but within the level of discrepancy expected due to differences in infill geometry. Note that conductivity can differ by a factor of 5.2 with different line infill patterns, see^27^. The model of conductivity for a given infill setting was thus

4 $${\rho }_{(S/m)}=0.0548\cdot infill-0.0432.$$

The residuals of the conductivity versus infill setting model were used to determine how many distinct levels of resistivity could be manufactured, i.e., the manufacturing resolution. For the model given in (4), the residuals were obtained by subtracting the model-predicted conductivity from the observed conductivity. Using the formula $stdev= \sqrt{\sum {\left(x-\overline{x }\right)}^{2}/\left(n-1\right)}$, where $x$ a single residual, $\overline{x }$ is the mean of the residuals, and $n$ is the number of residuals, the standard deviation of the residuals was calculated to be 0.168%. Assuming that the residuals follow a normal distribution and are uncorrelated with infill percentage setting, we can divide the desired infill range by a selected multiple of the standard deviation. Using an allowable range of 10% to 100% infill, and a factor of 2σ (equating to a 95% confidence interval), the number of distinct manufacturable divisions was calculated as

5 $${N}_{divisions}=15.$$

### Manufacturing uncertainty

**Figure 9:**
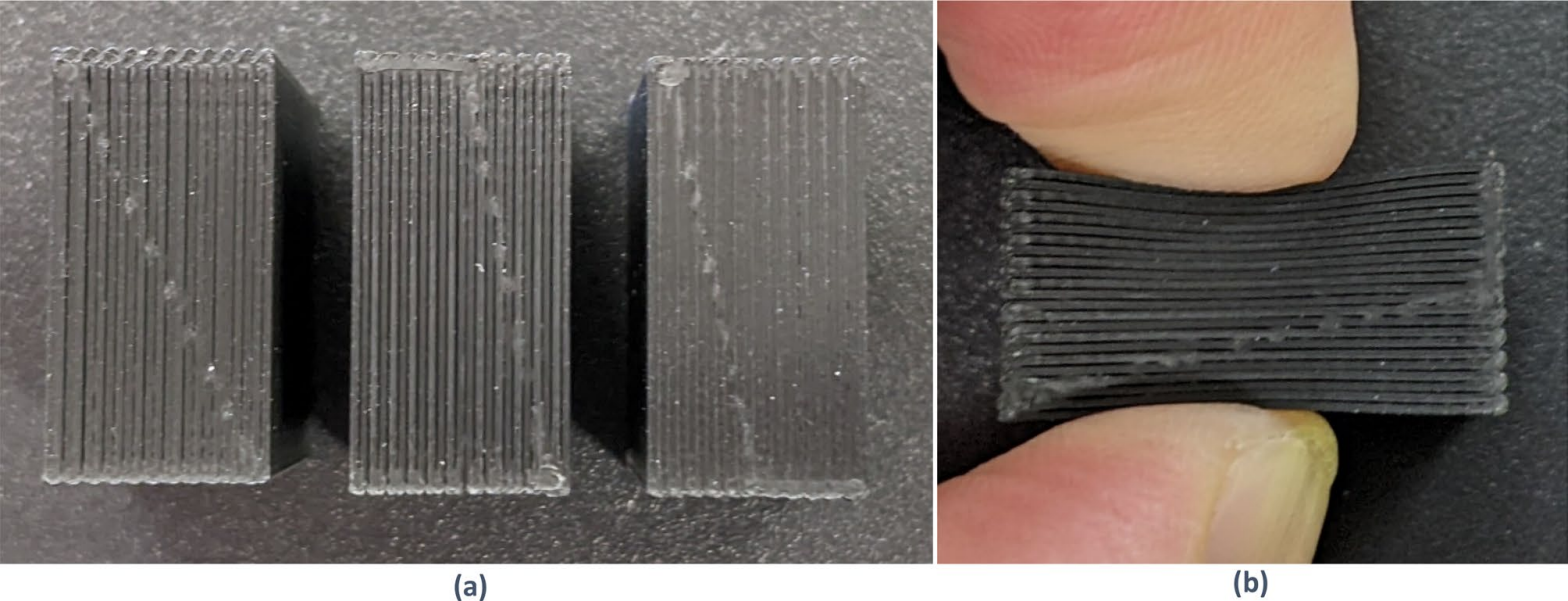
Samples printed with 100% infill. (**a**) Left—Vertical lines of filament have many gaps, with some bonds between them. Center—most vertial lines are separated. Right—almost complete bonding between vertical lines. (**b**) Deformation in (**a**) center sample with light fingertip pressure.

Significant variability in samples that were printed with the conductive material was observed. This is well illustrated by the samples in Fig. 9. The left image shows three different samples printed with identical settings. The samples were printed with 100% infill, with infill lines oriented exclusively in the vertical direction as viewed in the image. In the first and second samples pictured, significant space was visible between the printed filaments. The second sample was porous enough that it could be easily deformed by fingertip pressure, while the third was solid as designed.

The solid sample from Fig. 9, the third pictured in the left image, was printed directly after cleaning the print head on the Ultimaker S3, while the two with voids were printed after several other samples. There is a standard process for cleaning the print head of the Ultimaker S3, but it is only intended to be used in case of an abnormal blockage, not as part of routine maintenance. During each cleaning procedure, black residue was found in the interior of the print head nozzle.

**Figure 10:**
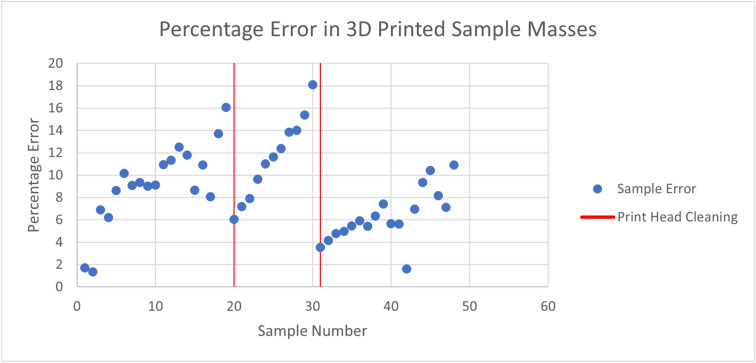
Percentage error in 3D printed sample masses for samples printed with Protopasta conductive PLA. Occasions of cleaning the 3D printer print head are indicated.

The observed variability has the potential to affect the achievable resistivity resolution of the phantom, so it is desirable to characterize and/or mitigate this effect. The variability was quantified by calculating percentage error in sample masses. Error was calculated by weighing each sample and comparing the measured mass to that calculated by the 3D printing software, which takes into account material density and assumes an ideal printed geometry. Figure 10 shows the percentage error versus sample number in order of printing. Print head cleanings are marked with a vertical line. It was evident that error decreased after print head cleaning and increased thereafter with successive prints. Samples 22–30 were printed sequentially in one print, demonstrating that error increases not just between prints, but during prints.

To confirm that the observed increase in print error was a property distinct to the conductive 3D printing filament, a set of nine samples in standard non-conductive PLA was printed. The average percentage error for the conductive material was 8.68%, with a standard deviation of 3.74%. The average percentage error for non-conductive material was 7.39%, with a standard deviation of 0.41%.

The standard deviation of percentage error for non-conductive samples was therefore nearly ten times lower than that of the conductive samples. Error did not increase with sequential samples. Interestingly, average error was rather high, at 7.39%. This indicates either a discrepancy between true density and nominal density of the material, or an inability of the printer to meet the designed characteristics. In either case, the absolute value of the percentage error is of much lower concern than variability in error for the current purposes, since designed resistivity is subject to calibration, which would account for mean error.

Unfortunately, although it was observed that error decreased after cleaning and increased thereafter, it appeared that the level of decrease and rate of increase were not consistent. Figure 10 appears to show increase in error with successive prints after print head cleaning, but the level of decrease with cleaning, and the rate of increase thereafter were not consistent across cleanings. Further work should be conducted to mitigate this effect. Changing either print settings or the material used could prove helpful.

It is not known exactly what effect the error in printed mass has on the resistance of a sample, but the variability in mass error of the infill percentage samples was measured at 3.73 (standard deviations), similar to the global variability figure, so we can be confident that the maximum effect is encompassed by the conductivity residuals in the existing linear model of conductivity versus infill percentage.

## Phantom prototype

A circular phantom was designed to act as a proof of concept for the proposed phantom method. For simplicity of proof of concept this phantom was functionally two dimensional. The phantom consisted of a circle 100 mm in diameter, and 20 mm in height. The diameter allowed it to be printed using the Ultimaker S3. The height was sufficient for the gyroid pattern to repeat several times. The phantom featured a circular inclusion with 20 mm diameter, which was 20% of the exterior diameter. This should be easily resolved using a 16 electrode EIT system. Brass electrodes were glued to the phantom using silver epoxy. The circular clamp that was used to hold the electrodes in place while gluing was also used to hold the electrode wires from the EIT device against the brass electrodes. The dimensional accuracy of the printed phantom was measured using a set of digital callipers. The maximum error in diameter of the inclusion was found to be 0.5 mm, and the positional error of the inclusion was found to be 0.1 mm.

**Figure 11:**
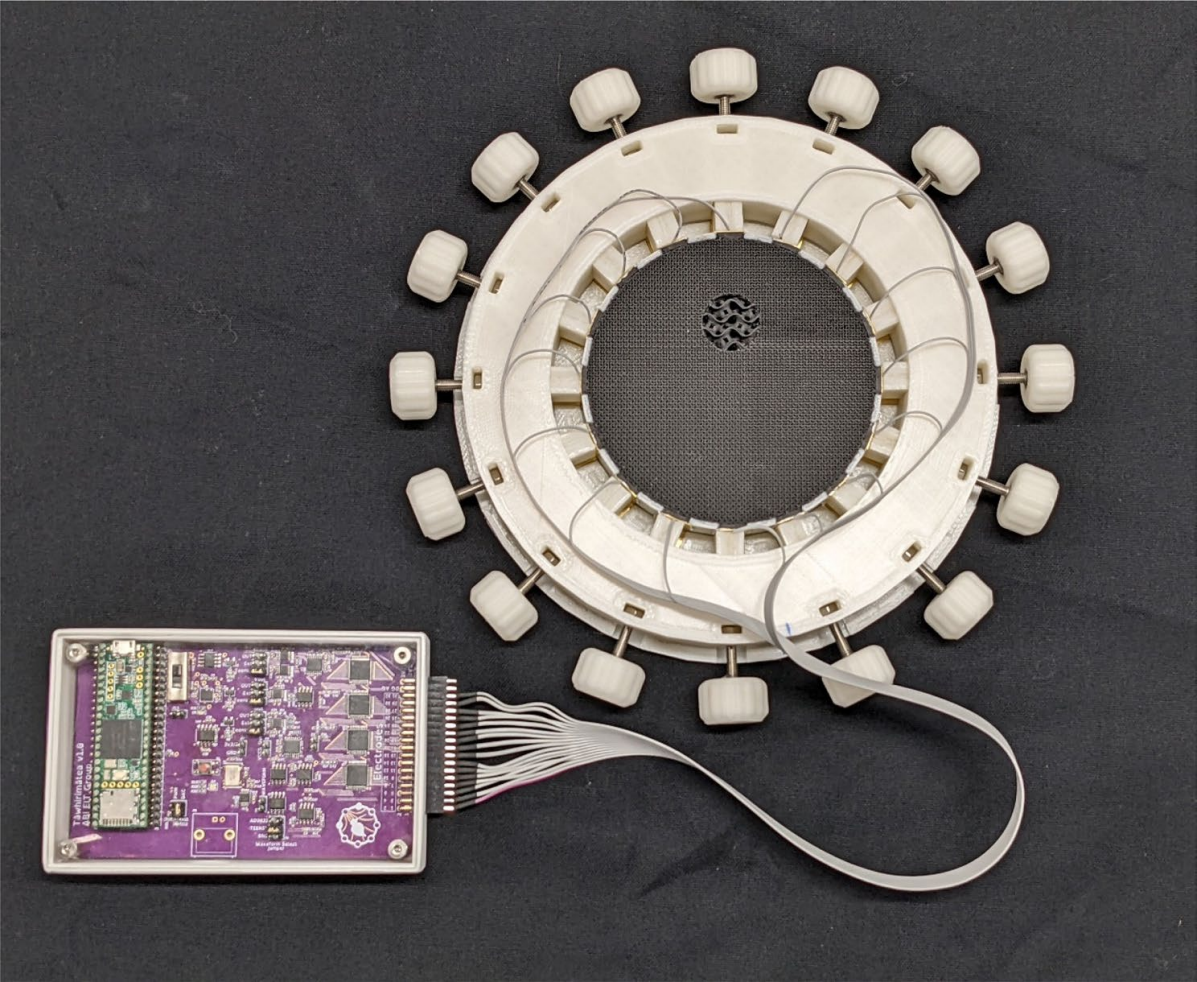
Phantom prototype with circular clamp, connected to custom made EIT device.

By Eq. (4), for a target of representing 10 Ωm lungs and 2 Ωm surrounding tissue, the required infill setting would be 2.6% and 9.9% respectively. However, such low percentage infills result in coarse geometry, particularly in the target region where the gyroid pattern would not be fully complete. In order to achieve sufficient homogeneity in the phantom infill geometry, a minimum of 10% infill was set. Given the limit of maintaining the same target to background conductivity ratio, the infill percentage of the inclusion was set to 11% and that of the background to was set to 55% (yielding an inclusion resistivity of 1.79 Ωm and a background of 0.34 Ωm). This phantom prototype is shown in Fig. 11.

### Prototype reconstruction analysis

To demonstrate the usage of the prototype phantom, a sample analysis was conducted. A reconstruction of physical EIT measurements taken using the phantom was compared to a reconstruction of simulated EIT measurements computed using a forward model.

EIT measurements were performed on the phantom using our custom EIT Device. The protocol is shown in Table 7:

**Table 7:** EIT device protocol.

Excitation	60 µA, 24 kHz
Drive	Differential, Skip 2
Measurement	Differential, Adjacent

For reconstruction, a triangular mesh was used with 2000 elements. Electrodes positions were specified at equally spaced intervals around the circumference. The pyEIT software package was used to perform reconstruction^28^. Specifically, a one-step Gauss–Newton reconstruction algorithm was used (see^29^) of the form:

$$\widehat{x}= {\left({J}^{T}J+\lambda R\right)}^{-1}{J}^{T}y,$$

$$R={\left[{J}^{T}J\right]}_{i,i}^{p},$$

where $\widehat{x}$ Is an estimate of the values of the elements in the image, $y$ Is the difference between two sets of measured voltages, $J$ Is the Jacobian matrix, $\lambda$ Is the regularization hyperparameter that controls the degree of regularization, $R$ Is the regularization matrix, made up of diagonal elements of ${J}^{T}J$, scaled by the exponent $p$

Note, these methods are not designed to reconstruct accurate conductivity values, but to produce useful images. The reconstruction was tuned using the hyperparameter *λ* which controls the trade-off between resolution and noise attenuation in the reconstructed image. We heuristically selected the *λ* value as 0.05 by choosing the value that produced the image most visually similar to the phantom, and the *p* parameter was set to the default 0.5, a compromise between pushing noise to the boundary and to the centre. The required “background” measurement for the reconstruction algorithm was provided by averaging together groups of measurements in the measurement frame corresponding to equal relative distances from the excitation electrodes.

For comparison, a simulation of the phantom was produced using pyEIT’s forward model solver. The simulation mesh was designed to match the ideal dimensions of the phantom.

**Figure 12:**
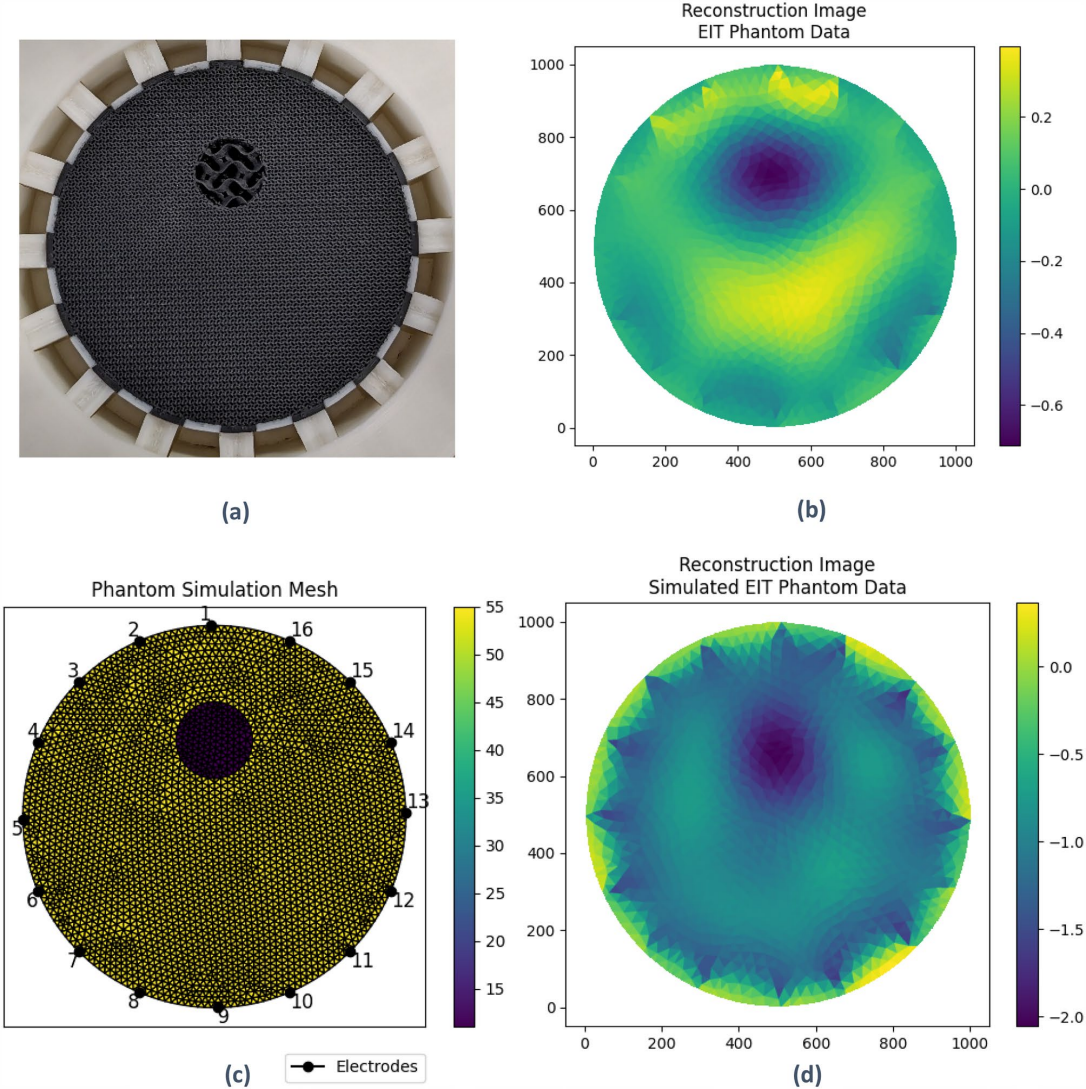
Reconstructed images versus source objects. (**a**) Phantom Prototype. (**b**) Reconstruction of phantom data. (**c**) Phantom simulation mesh. (**d**) Reconstruction of simulated phantom data.

Figure 12 shows the reconstruction images for both the physical and simulated datasets, alongside the physical phantom and the phantom simulation mesh. The reconstructions of the physical and simulated data were substantially similar, showing a dark spot in the upper central region. A noted difference was a biasing of the target spot towards the centre in the simulated reconstruction. Triangular artifacts at the electrodes were visible behind the target spot in the physical reconstruction, whereas they were apparent all around the image in the simulated reconstruction.

Using the phantom simulation mesh as a reference target, we computed position error and shape deformation as defined by Adler et al.^30^. Position error of the reconstructed image was 5.5% of the image diameter, and shape deformation was 26% (referring to the proportion of pixels in the thresholded image lying outside the bounds of a circle of equivalent area). To put the dimensional accuracy of the printed phantom into these terms, the measured position error of the inclusion was 0.1% and the equivalent shape deformation was approximately 0.8%. This indicates that the dimensional accuracy of the phantom is sufficient to assess the performance of the EIT hardware and software combination used for this study (Supplementary file 1).

## Discussion

A novel 3D printing method for constructing phantoms for EIT has been proposed. The new method allows a solid state EIT phantom with an arbitrary interior resistivity distribution to be manufactured automatically. The benefits of the new type of phantom over existing types have been demonstrated in several areas:

1. *Resistivity Resolution*—It has been shown that varying the 3D printing infill percentage parameter allows control of the relative resistivity of different sections of the phantom. Despite some manufacturing uncertainty, 15 distinct levels of resistivity can be printed in a single phantom. This eliminates the need to select different materials for each level of resistivity required in a phantom.
2. *Spatial Resolution*—3D printing technology allows arbitrary shapes to be manufactured with high spatial resolution. The Ultimaker S3 used in this study reports a resolution of 6.9 µm, 6.9 µm, and 2.5 µm in the *X*, *Y*, and *Z* coordinate axis directions respectively, far higher than the effective resolution of discrete element phantoms which previously were the only solid-state phantom type available.
3. *Stability*—The phantom is manufactured from a mixture of PLA and carbon black, two highly stable materials that are not subject to significant degradation in a laboratory environment. Further studies could be performed to determine if drift over time in the resistivity of the phantom can be measured. This behaviour can be contrasted to that of a salt bath, where the effects of evaporation over a period of hours can change the electrical characteristics of a phantom.
4. *Manufacturability in 3D*—The 3D printing technology makes manufacture of arbitrary shape in two and three dimensions trivial. Intricate details can be introduced into a phantom with no manufacturing penalty.

Overall, the 3D printed phantom can fill a role in EIT development that neither existing type of phantom has yet filled. Its combination of stability and resolution (both spatial and in resistivity) allow it to provide reference that is well known and constant across measurements, while still sufficiently detailed to be a useful approximation of real imaging subjects. While only a single inclusion was demonstrated here, the approach is sufficiently flexible to include multiple inclusions. This complexity is particularly necessary in studying EIT techniques that take into account geometric characteristics.

With the measured relationship between conductivity and infill percentage setting, it was noted that to use the required infill setting to achieve a physiologically representative resistivity would result in prohibitively coarse infill geometry. To rectify this, a material of higher bulk resistivity should be selected if the goal is to replicate the resistivity of the physiological system.

One aspect of the phantom not characterized in this study is the electrical anisotropy due to the 3D printing process. When 3D printing conductive materials, the non-ideal fusing between deposited lines of filament results in a higher resistivity across lines than along them. To partially mitigate this effect, we have used the gyroid infill pattern, which is triply periodic, reducing the overall difference in resistance between the *X* and *Y* axes. However, this pattern is not continuously rotationally symmetrical, so will exhibit some difference in resistance when measured from different angles. Additionally, the 3D printing process features no lines of filament oriented in the *Z* axis direction, which will result in differing resistance measurements for an object of equal dimensions measured in the *X* or *Y* axes and the *Z* axis. These effects should be characterized in future studies. It is encouraging to note, however, that a reasonable reconstruction was achieved using the prototype phantom without accounting for any effects of anisotropy.

In this study, only the real component of impedance was measured (i.e., complex impedance was not measured). Interesting avenues for future study would include measuring the reactance of the 3D printed material, investigating whether this is affected by the geometry of the infill pattern, and measuring complex impedance of the material at a range of excitation frequencies.

Future studies should expand on the 3D nature of the phantom. Though the manufacturing method of the phantom is intrinsically 3D, a phantom with three-dimensional geometry has not yet been demonstrated. Such a phantom would greatly improve the approximation of the phantom to real imaging subjects, with a minimal increase in manufacturing complexity.

### Supplementary Information

Supplementary Information 1.

Supplementary Information 2.

Supplementary Information 3.

Supplementary Information 4.

Supplementary Information 5.

Supplementary Information 6.

Supplementary Information 7.

Supplementary Information 8.

Supplementary Information 9.

## Data Availability

All data generated or analysed during this study are included in this published article (and its supplementary information files).
